# Screening of X-ray responsive substances for the next generation of radiosensitizers

**DOI:** 10.1038/s41598-019-54649-2

**Published:** 2019-12-03

**Authors:** Akihiro Moriyama, Takema Hasegawa, Lei Jiang, Hitoshi Iwahashi, Takashi Mori, Junko Takahashi

**Affiliations:** 1 The United Graduate School of Agricultural Science, Gifu University, 1-1 Yanagido, Gifu, Gifu 501-1193 Japan; 2 Molecular Composite Medicine Research Group, Biomedical Research Institute, National Institute of Advanced Industrial Science and Technology (AIST), 1-1-1 Higashi, Tsukuba, Ibaraki 305-8566 Japan; 3 Animal medical Center, Gifu University, 1-1 Yanagido, Gifu, Gifu 501-1193 Japan

**Keywords:** Radiotherapy, Photobiology, Reaction mechanisms, Radiotherapy

## Abstract

X-ray responsivity resulting in the generation of reactive oxygen species (ROS) was investigated in 9600 organic compounds that were selected by considering their structural diversity. We focused on superoxides that were primarily detected using dihydroethidium (DHE) and hydroxyl radicals, that were identified fluorometrically using 3’-(p-aminophenyl) Fluorescein (APF). Many organic compounds were discovered that responded to the DHE and/or APF assay using X-ray irradiation. These results suggest that some of these organic compounds emit either superoxides or hydroxyl radicals whereas others emit both under the influence of X-ray irradiation. The response of the derivatives of a hit compound with a partial change in the structure was also investigated. The products produced from DHE by X-ray irradiation were identified by HPLC to confirm the integrity of the process. Although, the reactions were suppressed by the superoxide dismutase (SOD), not only 2-hydroxyethidium (2-OH-E^+^), but also ethidium (E^+^) were detected. The results suggest that apart from a direct reaction, an indirect reaction may occur between DHE and the superoxides. Although X-ray responsiveness could not be inferred due to the molecular complexity of the investigated compounds, delineation of these reactions will facilitate the development of the next generation of radiosensitizers.

## Introduction

Radiotherapy is commonly used as part of the treatment protocol in cancer therapy. However, acute toxicity and potential long-term adverse effects often limit the dose of radiation to levels that are insufficient to effectively treat tumors. The use of radiosensitizers that enhance radiation effects in tumors can improve therapeutic efficacy. However, to date, no radiosensitizers that directly enhance the effects of X-ray by physical or physicochemical reactions for ionizing radiation with clinical benefits have been discovered. Recently, there was a report that ROS were generated when protoporphyrin IX (PpIX) was irradiated by X-ray^1^. Exogenous porphyrin selectively accumulates in cancer cells and generate ROS to induce localized cellular damage when irradiated with light. Using this process, photodynamic therapy has been developed as a treatment for cancer^2,3^. However, ROS production by PpIX in response to X-ray radiation can potentially be exploited in the development of a radiosensitizer for radiodynamics therapy^4,5^. To increase the possibility and applicability of radiodynamic therapy, plant-derived organic compound libraries were screened to identify potential X-ray responsive substances (XRS) that are non-toxic to animals. As a result, several organic substances, including a flavonoid called luteolin, were found to be XRS^6^. Owing to their molecular complexity, the XRS responsiveness of these substances could not be deduced based only on their structural analysis.

Radiation causes cleavage of the main chain in DNA and damage to the base. In low LET radiation such as X-rays and γ rays, apart from cleavage of the DNA chain by direct action, damage to various bases owing to indirect action is caused^7^. This indirect action involves ROS generated by X-rays. ROS destroyed the redox balance and resulting in oxidative stress that affects some cellular molecules and induce DNA damage^8^. Furthermore, oxidative DNA damage prior to replication may lead to mutation, replication errors, and even cell death if not repaired correctly^9,10^.

In this study, we focused on the chemical interactions between low molecular weight organic compounds and X-ray irradiation, particularly ROS generation, and searched for X-ray responsive substances that can generate ROS from 9600 organic compounds selected based on their structural diversity. We attempted to associate or correlate the molecular characteristics of organic substances to their X-ray responsiveness. Our study will contribute to a fundamental understanding of the chemical reactions between organic compounds and X-rays irradiation to facilitate the development of new radiosensitizers.

## Results

### XRS responsiveness of quercetin

**Figure 1:**
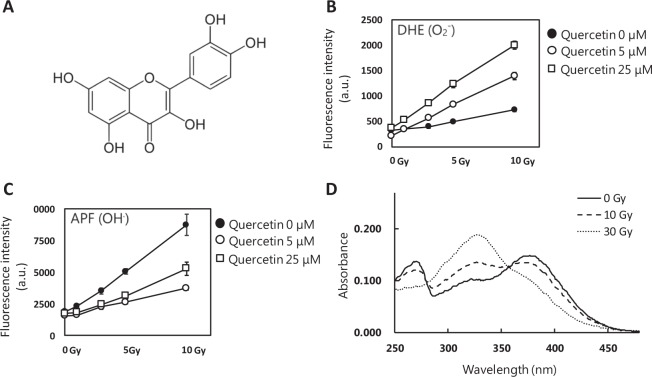
Properties and X-ray responsiveness of quercetin. (**A**) Structural formula of quercetin. (**B**,**C**) Fluorescence intensities of ROS detection reagents irradiated by X-ray (0, 1, 3, 5, and 10 Gy) with quercetin (5 µM or 25 µM), (**B**) 50 µM DHE, (**C**) 5 µM APF, and (**D**) Spectra of quercetin before or after X-ray irradiation (10 or 30 Gy).

Firstly, we investigated the XRS responsiveness of quercetin (Fig. 1A), which is a flavonoid similar to luteolin^6^. We focused on two types of free radical, superoxides and hydroxyl radicals. Both the superoxide and the hydroxyl radical were detected fluorometrically. The superoxides were measured using a DHE assay system^11–13^, while hydroxyl radicals were measured using an APF assay system^14^. In this study, the XRS responsiveness of target compounds was determined based on the fluorescence intensities of these detection reagents. The influence of ROS and the other radicals on the detection reagent were also considered. However, we regard it as the characteristics of the organic compound. In summary, the screened target is a compound that affects the fluorescence intensity of the detection reagent based on a radical reaction induced by exposure to X-ray irradiation.

After quercetin was dissolved in DMSO, it was diluted with PBS (Phosphate buffered salts) to a concentration of 5 µM and 25 µM (0.25% DMSO) because the library of 9800 compounds for screening was dissolved in DMSO. Given that DMSO is a quencher of ROS, its concentration was kept to a minimum of 0.25%. The DMSO concentration was controlled to remain constant for each condition. The solutions were irradiated with X-ray at 3, 5, and 10 Gy using Faxitron CP-160 irradiator (Faxitron X-ray Corporation). DHE with a final concentration of 50 µM was added to the irradiated X-ray quercetin samples. The samples were excited at 485 nm and the fluorescence was detected at 610 nm (Infiniti M200, TECAN). APF with a final concentration of 5 µM was mixed with the irradiated quercetin samples. The samples were then excited at 480 nm and fluorescence was detected at 520 nm. Consequently, in the DHE assay system, the slope of the fluorescence intensity of 0 µM, 5 µM, and 25 µM of quercetin was 42, 119, and 165, respectively. In the APF assay system, it was 696, 226, and 363 respectively. As such, for DHE, the ratio of the slope was 2.9 and 4 times larger at 5 µM and 25 µM, respectively (Fig. 1B) compared to the control. However, the ratio of the slope was 0.3 and 0.5 times lower for APF at 5 µM or 25 µM, respectively (Fig. 1C). Prior to screening for X-ray responsiveness, the degree of response was estimated under different irradiation dose rates from 1 Gy/min to 5 Gy/min using quercetin and PpIX, as lead compounds. We were able to confirm the same degree of response under the same total dose conditions. Next, spectral measurements were performed on the quercetin samples that were irradiated at 0 Gy, 10 Gy, and 30 Gy to determine whether there was any associated structural change. As a result, it was determined that there is a shift in the peak wavelength in the vicinity of 370 nm to a lower wavelength after irradiation (Fig. 1D).

In our study, XRS responsiveness of quercetin was highly reproducible. The results of previous studies indicate that PpIX generates both superoxides and hydroxyl radicals with very low reproducibility. Hence, we decided to use quercetin XRS responsiveness as a positive control for the DHE and APF assay.

### Screening of XRS

**Figure 2:**
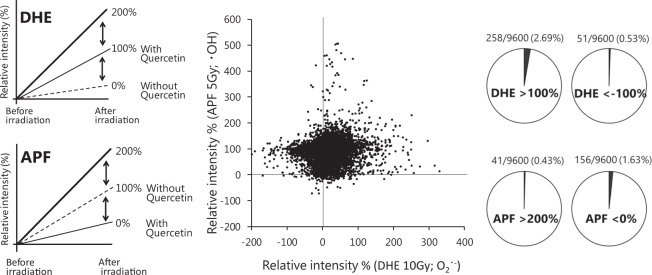
Relative fluorescence intensity of DHE and APF with each target compound irradiated by X-rays. 5 µM of each compound was irradiated with 50 µM DHE (O_2_^∙^^−^assay) or 5 µM APF(∙OH assay) at 5 or 10 Gy. The relative fluorescence intensity (%) was calculated in each case, DHE assay: intensity of 5 µM quercetin 100%, without quercetin 0%, APF assay: intensity of 5 µM quercetin 0%, without quercetin 100%.

We investigated the X-ray responsiveness of the compound library (drug discovery initiative (DDI), the University of Tokyo) based on the fluorescence of DHE or APF after irradiation as an index (Fig. 2). In this library, 9600 organic compounds were selected considering factors such as their structural diversity and for both analogous compounds and completely random compounds with reported activity. In the library, the minimum molecular weight is 122, the maximum is 614, and the average molecular weight is approximately 320. Each compound was adjusted to a final concentration of 5 µM, 0.25% DMSO, irradiated with 10 Gy for the detection of superoxides using DHE or 5 Gy for the detection of hydroxyl radicals using APF, and the fluorescence intensity after irradiation was measured. Given that the doses used for radiotherapy are 1.8–2 Gy for fractionated irradiation and 17 Gy for stereotaxic radiosurgery, we used 5 and 10 Gy for the screening and thus the dose levels are the same as those used for radiotherapy.

In the DHE assay, 5 µM quercetin was used as a positive control (100%) and the absence of quercetin was used as a negative control (0%). In total, 258 chemicals out of 9600 candidates exhibited responses that were above that of the positive control. In the APF assay, it was observed that quercetin response was much lower than the absence of quercetin (Fig. 1C). Hence, the absence of quercetin and 5 µM quercetin were used as a positive control (100%) and negative control (0%), respectively. Out of 9600 candidates, 41 compounds exceeded 200% that of the positive control (without quercetin). It was surprising to identify so many chemicals that could release high levels of superoxides and/or hydroxyl radicals in response to X-ray irradiation (Fig. 2).

### HPLC identification of the products formed from DHE or APF by X-ray irradiation

**Figure 3:**
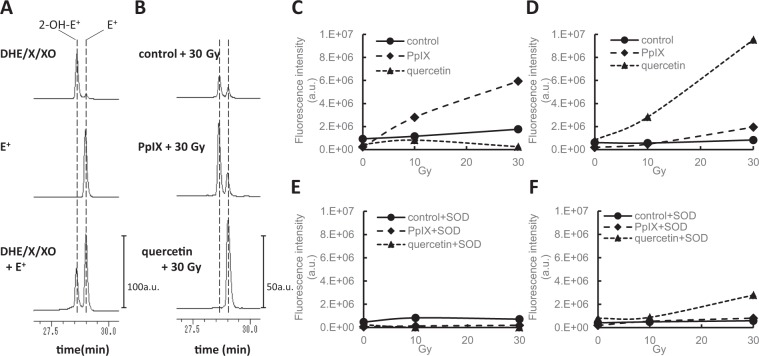
HPLC identification of the products formed from DHE by X-ray irradiation.(**A**) HPLC chromatograms of the products formed from oxidation of DHE by the X/XO system or ethidium. (**B**) HPLC chromatograms of the products formed from DHE with PpIX or quercetin by X-ray irradiation. (**C**) The irradiation dose-dependent effect of the product, 2-OH-E^+^, formed from DHE. (**D**) The irradiation dose-dependent effect of E^+^ formed from DHE. (**E**) The effect of SOD on the production of 2-OH-E^+^. (**F**) The effect of SOD on the production of E^+^. Fluorescence detection at 510 nm (excitation) and 595 nm (emission) was used to monitor the products formed from the oxidation of DHE.

**Figure 4:**
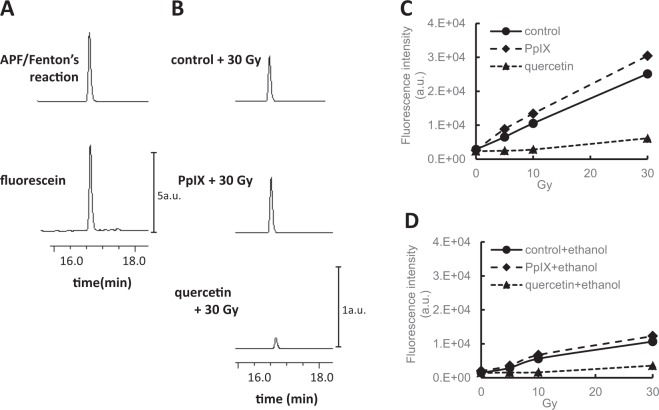
HPLC identification of the product formed from APF by X-ray irradiation.(**A**) HPLC chromatograms of the product formed from oxidation of APF by Fenton’s reaction system. (**B**) HPLC chromatograms of the product formed from APF with PpIX or quercetin by X-ray irradiation. (**C**) The irradiation dose-dependent effect of the product formed from APF. (**D**) The effect of ethanol on the product formed from APF. Fluorescence detection at 480 nm (excitation) and 520 nm (emission) was used to monitor the product formed from the oxidation of APF.

DHE and APF are used as probes for ROS detection. To confirm the integrity of our methodology based on these probes, the products produced from DHE or APF under the influence of X-ray irradiation was confirmed by HPLC. The dominant reaction product of DHE and the superoxide is 2-hydroxyethidium (2-OH-E^+^). However, ethidium (E^+^) is also produced and can be separated by HPLC as described previously^12,13^. Figure 3A depicts chromatograms of the oxidation products formed from DHE and the superoxides. Hypoxanthine/xanthine oxidase (HX/XO) was used to generate the latter. The product, 2-OH-E^+^, formed from DHE and HX/XO can be distinguished from E^+^. Figure 3B shows chromatograms of the products of DHE and PpIX, or quercetin irradiated with 30 Gy X-rays. PBS with 0.25% DMSO was used as a control. PpIX enhances 2-OH-E^+^ production, whereas quercetin enhances E^+^ production in an X-ray-dose-dependent manner (Fig. 4C,D). In both cases, generation was suppressed in the presence of SOD (7.5 units/ml) (Fig. 4E,F).

The reaction product of APF and the hydroxyl radicals is fluorescein, as described previously^14^. Figure 4A depicts the chromatograms of the oxidation products formed from APF and the hydroxyl radicals. Fenton’s reaction was used to generate hydroxyl radicals. The product, fluorescein, that is formed from APF and iron (II) perchlorate/hydrogen peroxide can be identified. Figure 4B shows the chromatograms of the products of APF and PpIX, or quercetin irradiated with 30 Gy X-rays. PBS with 0.25% DMSO was used as a control. PpIX enhances the production of fluorescein with X-rays in a dose-dependent manner (Fig. 4C), whereas quercetin suppresses the production. The generation of fluorescein was suppressed in the presence of 10% ethanol (Fig. 4D).

### Grouping of hit compounds based on the relative responsiveness

**Figure 5:**
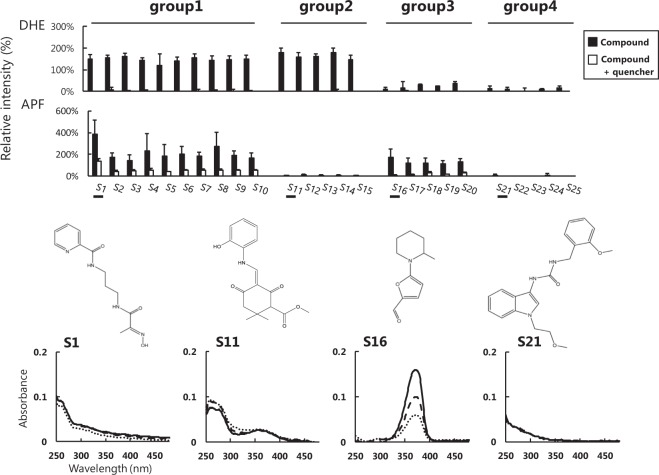
Grouping of hit compounds based on the relative fluorescence intensity of ROS detection regents using X-rays. Group1: Both DHE and APF high, Group2: DHE high (quercetin type), Group3: APF high, Group4: Both DHE and APF low. Each Compound was irradiated with X-rays (DHE-10Gy, APF-5Gy) with detection reagents in the absence or presence of each ROS quencher (DHE; 7.5 units/ml SOD, APF; 10% ethanol).

It was determined that the hit compounds identified through screening to date generate a different balance of the type and amount of ROS. Hit compounds were grouped by differences in their DHE and APF responses. The structural formulas of the grouped hit compounds are shown in Fig. S1. Based on X-ray responses, those that emit both superoxide and hydroxyl radicals are group 1 and those like quercetin that generate superoxides but eliminate hydroxyl radicals are categorized as group 2. Some compounds assigned to group 2 have a structure similar to quercetin (Fig. S2). In addition, there is also a type that only generates hydroxyl radicals (group 3). Group 4 includes compounds that substantially eliminate ROS, particularly hydroxyl radical when subjected to X-ray irradiation. For compounds with characteristic responses in each group, an X-ray response test was conducted under the condition with a ROS-specific quencher (Fig. 5). SOD (final concentration 7.5 units/ml) was added to the DHE assay as the quencher of the superoxide or ethanol^15^ was added (final concentration 10%) as the quencher of the hydroxyl radicals in the APF assay. From the results, the reproducibility of the X-ray response and generation of a specific ROS were confirmed (Fig. 5). Catalase^16^ was then added (final concentration of 100 units/ml) as a quencher in the APF assay to investigate the origin of the hydroxyl radical (Fig. S3). However, catalase did not quench the APF’s response. Therefore, the hydroxyl radical is not considered to be derived from superoxide via H_2_O_2_. Spectral measurements were also performed on hit compounds that were X-ray irradiated at 0 Gy, 10 Gy, and 30 Gy to determine whether the irradiation process resulted in structural changes (Fig. S1).

### X-ray responsive activity of the derivatives of each hit compound

**Figure 6:**
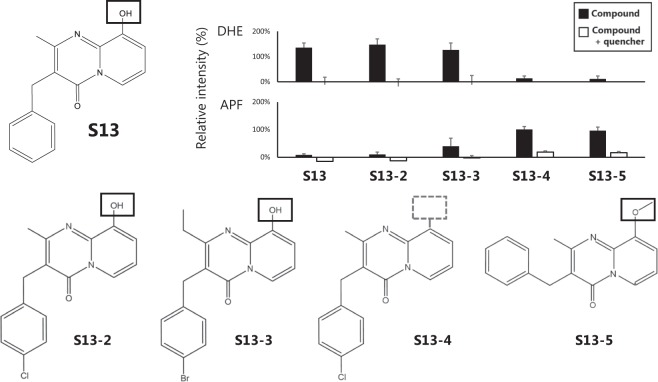
Relative fluorescence intensity of DHE and APF with each similar compound irradiated using X-rays. 5 µM of each compound was irradiated with 50 µM DHE (O_2_^∙−^ assay) or 5 µM APF(∙OH assay) at 5 or 10 Gy. Each Compound was irradiated with X-rays (DHE-10Gy, APF-5Gy) with detection reagents in the absence or presence of each ROS quencher (DHE; 7.5 units/ml SOD, APF; 10% ethanol).

We investigated the variation of the X-ray response with slight changes in some of the hit compounds. The X-ray responsiveness of the derivatives of each compound was measured using 50 µM DHE (10 Gy), 5 µM APF (5 Gy) after irradiation under the same conditions (5 µM, 0.25% DMSO). No significant difference in the response activity was confirmed even in comparison with the S13 compound and S13-2 for which Cl-residues were added to the terminal of S13 or S13-3 for which a Br-residue was added, and the carbon chain was further extended by one. However, in the case of S13-4 for which the OH group was removed from S13-2, low DHE fluorescence activity was observed and superoxides were not generated. For derivatives with an OH group, superoxides are generated and the hydroxyl radicals are eliminated (S13-1, 2, 3). However, the absence of the OH group reduces the activity so that with respect to S13, the activity depends on the presence of this group (Fig. 6). In addition, no activity was observed in S13-5, which had no OH groups and had different positions of the six-membered ring. In this way, it was determined that there are compounds that slightly differ in structure that significantly influence the activity. With respect to the S-13 compound, a decrease in the peak of the absorbance spectrum (=bleaching phenomenon) subsequent to X-ray irradiation was confirmed only in S13, S13-2, and S13-3 compounds that generate superoxides (Fig. S4).

### Effect of cell viability in irradiated XRS – treated B16/BL6 cells

**Figure 7:**
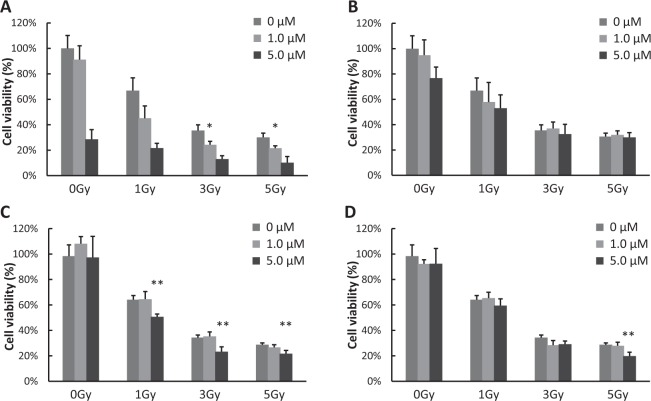
Viability of B16-BL6 cells treated with PpIX, quercetin, or typical XRS and X-ray exposure. Cell viability at 72 h post-irradiation (**A**) PpIX treatment, (**B**) quercetin, (**C**) S1, and (**D**) S6. PpIX, quercetin, S1 or S6 was added 3 h prior to X-ray irradiation at concentrations of 1 µM, 5 µM for each compound, and irradiated at a dose of 0, 1, 3, and 5 Gy. Statistical significance relative to the experiment performed without X-ray-responsive substances (XRS) at the same radiation dose is represented by (*p < 0.05, *p < 0.01).

To determine the effects of PpIX, quercetin, S1, or S6, and X-ray treatments on B16-BL6 cell viability, a WST-8 assay was performed (Fig. 7). B16-BL6 cells were incubated with increasing concentrations of PpIX or quercetin for 3 h prior to irradiation. PpIX, quercetin, S1 or S6 -treated cells were subjected to increasing levels of X-ray exposure and the cell viability was then analyzed at 72 h post-irradiation. The percent of survival was expressed with reference to non-irradiated control cells. Cell viability was decreased to increase the irradiation dose without using chemicals. It was determined that the viability of cells decreased as the PpIX, S1, or S6 concentration and X-ray dose increased compared to the case without XRS (Fig. 7A,C,D). In the case of quercetin, cell viability decreased in a similar manner to the case without chemicals at all irradiation doses (Fig. 7B). Quercetin did not exhibit a damaging effect on cells, possibly because of the effect of the scavenging hydroxyl radicals.

## Discussion

To examine the integrity of our methodology using DHE and APF probes, the products from DHE or APF due to X-ray irradiation was confirmed by HPLC. 2-OH-E^+^ is the dominant product of the reaction between DHE and the superoxides^12^. However, E^+^ was observed for low fluxes of superoxides^13^. Therefore, the mechanisms of formation of E^+^ is thought to be via an indirect reaction between DHE and the superoxides. The dominant reaction product of DHE and the superoxides is 2-OH-E^+^, and it can be separated from E^+^ by HPLC as described previously^12,13^. We confirmed that the dominant product of DHE and PpIX was 2-OH-E^+^ using X-ray irradiation (Fig. 3B). The product increased in an X-ray-dose-dependent manner and is suppressed using SOD (Fig. 3C,E). In contrast, E^+^ was the dominant product of DHE and quercetin with X-ray irradiation (Fig. 3D). E^+^ increases in an X-ray dose-dependent manner, and is also suppressed by SOD (Fig. 3D,F). The reaction of DHE and quercetin under X-ray irradiation is clearly different from that of PpIX. However, the reaction is thought to be caused by the superoxides, because both reactions were suppressed by SOD. We established that confirmation via HPLC is necessary to identify the dominant product to identify the kind of reaction. Regarding APF, fluorescein is generated with hydroxyl radicals via Fenton’s reaction. We identified fluorescein via the reaction of APF and X-ray irradiation using HPLC. The amount of Fluorescein increased in an X-ray-dose-dependent manner. PpIX enhances it, whereas it is suppressed by ethanol. Thus, we concluded that APF is an indicator of the hydroxyl radicals in this study.

In this screening, many compounds that generated hydroxyl radicals were identified. Therefore, it is expected that new sensitizers will need to be developed. We assigned hit compounds to each group based on the results of the ROS detection assay. Hit compounds that emit both superoxide and hydroxyl radicals are group1 and those that generate superoxides but eliminate hydroxyl radicals are categorized as group 2. Those that emit only hydroxyl radicals are group 3. Group 4 includes compounds that substantially eliminate ROS, particularly hydroxyl radical when subjected to X-ray irradiation. In each group, some common points were confirmed from part of the structure, but almost compounds exhibited low commonality (Fig. S1). This means that the X-ray response of organic compounds has diversity and complexity that cannot be understood by only considering their structures. Cyclic structures and double bonds in the structure may contribute to a response but do not guarantee a response. APF fluorescence increased according to the X-ray irradiation dose regardless of the dissolved oxygen concentration. It is thought that APF reaction with surrounding H_2_O molecules probably accounts for this observation. DHE fluorescence increased according to the X-ray irradiation. The sensitizer and the concentration of dissolved oxygen amplify the DHE reaction (Fig. S5). Thus, the DHE reaction was affected by the surrounding O_2_ molecules. There is a possibility that such a phenomenon may be confirmed with other hit compounds and further study is needed.

The intracellular response of the sensitizer is influenced by cellular uptake and intracellular distribution. Hydroxyl radicals have very high reactivity, but owing to their short lifetimes, they often respond to cellular constituents at a rate close to the rate-limiting rate and cannot be damaged unless target molecules are located very close to the site of production. Regarding PpIX, it was previously reported that it facilitates the release of hydroxyl radicals by X-ray irradiation in the same experimental system^1^ (Fig. S6). PpIX is known to be easily taken up by cancer cells and has been confirmed to generate ROS in HeLa cells by X-ray irradiation using APF and DHE^17^. In addition, 5-aminolevulinic acid, which is a precursor of PpIX, is known to accumulate PpIX specifically in cancer cells, and is clinically used as a photosensitizer in photodynamic therapy for cancer treatment^2,3^. Visualization of intracellular 5-ALA-induced PpIX and ROS induced by ionizing irradiation in *in vitro* 9 L rat gliosarcoma cells has been reported^18^. DCF fluorescence generated by the oxidant sensitive probe 2’,7’-dichlorofluorescein diacetate (DCFD) has been shown to be predominantly co-localized with 5-ALA induced PpIX after ionizing irradiation. In this report, the viability of mouse melanoma B16-BL6 cells decreased according to the treatment with increasing PpIX, S1 or S6 concentration (Fig. 7A,C,D). The radio-sensitization effect of PpIX, S1, or S6 on the cells was shown. However, this effect is not particularly strong for a single irradiation. However, in radiotherapy, fractional irradiation typically ranges from 40 Gy to 60 Gy; 20–30 fractions are commonly applied in treatment regimens of patients, and the effect on cells is integrated with each irradiation. As a result, the use of XRS would likely have a large damaging effect on cancer cells. The cellular uptake or intracellular distribution of S1 or S6 has not been studied. Thus, these results are preliminary data, and further detailed examination is necessary. In the cells, the superoxides are decomposed by SOD, but excessive superoxides become hydroxyl radicals. They have very short half-lives of 10^−9^ seconds and 10^−6^ seconds, respectively. When they are generated near the target molecule, they function as a radiosensitizer because they are effective for cell damage^19^. Against this background, not only is the generation and the type of ROS important but also cellular uptake and intracellular distribution are critical for the development of sensitizers in the future.

Oxygen is a well-known radiosensitizer that acts via a mechanism of damage fixation based on its electron affinity. Small-molecule chemicals such as Nicotinamide, Clofibrate, 5-nitroimidazole, 5-fluorouracil, and gemcitabine that serve as oxygen generators or their mimics, are expected to also serve as radiosensitizers^20,21^. Some of them have proven to be clinically useful. Gold nanomaterials have shown good radiosensitizing effects in a variety of tumors with acceptable chemical stability, high biocompatibility, and low toxicity. Other high Z metal-based nanomaterials, such as silver nanoparticles and bimetallic nanoparticles, have also been investigated^20,22^. The problem with these agents is tumor selectivity. Otherwise, they may potentially be powerful radiosensitizers. However, there are compounds that are selectively taken up or produced in tumors. Photosensitizers, such as lezerphyrin or photophyrin which are porphyrin derivatives, or 5-aminolevulinic acid (5-ALA) which is a natural precursor of PpIX, are examples of these compounds. Photosensitizers facilitate selective accumulation in tumor cells. Subsequent excitation by laser beam causes the photosensitizer to assume a singlet state, which emits fluorescence upon returning to the ground state, accompanied by ROS production. A relatively new approach, the use of X-rays instead of UV-visible light for deeper penetration into tissue has been discussed^23^. The benefit of ALA mediated PpIX are considered due to Warburg effect. However, their uptake into cancer cells are depend on cancer cells characteristics and new candidates are required.

In our study, XRS responsiveness of quercetin was investigated and the results indicate that this compound releases superoxides in response to X-ray irradiation but quenches the hydroxyl radicals generated by x-ray and water response^24^. Quercetin is considered to be an antioxidant substance^25^. This reaction may be the basis for antioxidant compounds. Although the probability that this compound reacts directly and decomposes with APF or DHE and the fluorescence intensity is low, it cannot be completely eliminated at present. It can also be utilized as a radioprotective agent or active oxygen scavenger if it can eliminate ROS. Research and development on radioprotective agents have been conducted thus far. In this screening, we focused not only on the generation of ROS but also on the scavenging of ROS by organic compounds. For example, compounds belonging to group 4 exhibit a strong scavenging ability, particularly with respect to the hydroxyl radicals. In terms of the removal of ROS, not only the use as an antioxidant, but also the ability to specifically remove ROS during X-ray irradiation allows the possibility of serving as a new type of ROS scavenger. For example, vitamin E, vitamin C^26,27^ and epigallocatechin^28^ are known as antioxidant compounds. At the moment, it is not possible to determine whether a compound is an antioxidant that always captures and stabilizes radicals like these compounds or it only exhibits antioxidant behavior subsequent to X-ray irradiation. In consideration of the *in vivo* redox balance, the development of ROS generating or scavenging compounds that exhibit this behavior only after X-ray irradiation can greatly contribute to the improvement of treatment efficiency or the reduction of biological load.

Several photosensitizing compounds react with various ROS and it has been reported that they undergo photobleaching in this process^29–31^. For example, Nretinylidene-N-retinylethanolamine (A2E) known as a light absorbing substance has been confirmed to exhibit a decrease in its absorbance peak and a change of its structure after irradiation at 430 nm in a photooxidation reaction. It has been shown that singlet oxygen is involved in this photooxidation, and A2E acts as a sensitizer for generating singlet oxygen from triplet oxygen^23^. We suspected that the phenomenon called radio bleaching is similar this photobleaching is associated with the decrease of the absorbance peak in the hit compounds. When the absorption spectrum of hit compounds were measured after X-ray irradiation, some compounds exhibited strong bleaching level same as photobleaching. While some compounds showed a change in their spectrum. Others showed little change in their spectrum (Fig. S1). The possibility that it is involved in various ROS generation with structural change has been suggested by the change of waveform. Further understanding of the radiation response of organic compounds will be advanced by analysis of detailed chemical structure change using Mass spectrometry analysis

In conclusion, several X-ray responsive substances were identified by screening and their X-ray responses were found to be diverse. Although we have not yet elucidated the mechanism, we would like to clarify this aspect based on the structure and properties of the novel responsive substance discovered as part of this research. We found many compounds that exhibited XRS. The research field of organism-derived radiosensitizer is novel and in its infancy. To understand complex reactions, it is necessary to accumulate appropriate evidence to understand the mechanisms. Mass spectrometry analysis and different kind of probes may contribute to the advancement off this field. The elucidation of these X-ray responses will assist in the development of the next generation of radiosensitizers.

## Methods

### Materials

Quercetin, protoporphyrin IX (PpIX), dimethyl sulfoxide (DMSO) was purchased from Sigma-Aldrich, USA. Ethanol, superoxide dismutase (SOD), catalase, ethidium bromide, hypoxanthine, diethylenetriaminepentaacetic Acid (DTPA), trifluoroacetic acid, acetonitrile, iron(II) perchlorate, hydrogen peroxide, fluorescein, phosphoric acid, Dulbecco’s phosphate-buffered saline (D-PBS) were purchased from FUJIFILM Wako Pure Chemical Corporation., Japan. 3’-(p-aminophenyl) Fluorescein (APF) was purchased from Sekisui Medical Co. Ltd., Japan. Dihydroethidium DHE was purchased from Invitrogen, CA. Compound libraries (Core library; for pilot screening) were provided by drug discovery initiative (DDI), the University of Tokyo (https://www.ddi.u-tokyo.ac.jp/en/). WST-8 was purchased from Dojin Laboratoies, Japan. Xanthine oxidase was purchased from Calbiochem (USA).

### X-ray irradiation

X-ray was generated from an X-ray generator (Faxitron CP-160 irradiator, Faxitron X-ray Corporation, IL, USA). Irradiation periods were adjusted to 1.0, 5.0, 10.0, and 30.0 minutes to obtain the incident X-ray dose of 1.0, 5.0, 10.0 and 30.0 Gy, respectively.

### Measurement of ROS generation

A DHE assay was used to measure superoxide generation, while an APF assay was used to measure hydroxyl radical generation. The assays were performed in microplates and signals were detected using a microplate reader, Infinite M200, TECAN. DHE was mixed with the samples to a final concentration of 50 µM. The mixtures were excited as 485 nm and the fluorescence responses were detected at 610 nm. In APF assay, samples were mixed with APF to a final concentration of 5 µM. The mixtures were excited at 480 nm and fluorescence was detected at 520 nm.

To evaluate the effect of dissolved oxygen for ROS generation, N_2_, air, or O_2_ gas was bubbled through the reaction mixture. A volume of 1 mL quercetin at 5 µM and 50 µM DHE or 5 µM APF were placed in gas-tight 5 mL containers with a gas inlet and outlet port. After N_2_, air, or O_2_ gas was bubbled through the containers for 2 minutes, the inlet and outlet valves were shut off, and X-ray irradiation was immediately performed. Subsequently, each mixture with DHE was measured by microplate reader at Em: 485 nm and Ex: 610 nm, mixture with APF was measured at Em: 480 nm and Ex: 520 nm.

### HPLC analysis

The oxidation products of DHE or APF were separated using an HPLC (Shimadzu, Osaka, Japan) system equipped with fluorescence and UV detectors. The mobile phase was H_2_O/CH_3_CN and the stationary phase was a C_18_ reverse-phase column (Inertsil ODS-3, 250 × 4.6 mm, GL Sciences Inc., Tokyo, Japan). After the column was equilibrated with 10% CH_3_CN in 0.1% trifluoroacetic acid, 50 μL of the sample was injected into the HPLC system. The product formed from oxidation of DHE was separated via a linear increase in CH_3_CN concentration from 10% to 70% in 46 min at a flow rate of 0.5 mL/min, as described previously^12^. The fluorescence detection at 510 nm (excitation) and 595 nm (emission) was used to monitor these compounds. After the column was equilibrated with 10% CH_3_CN in 0.1% phosphoric acid, the products formed from oxidation of APF were separated via a linear increase in the CH_3_CN concentration from 10% to 70% in 25 min, at a flow rate of 1 mL/min. Fluorescence detection at 480 nm (excitation) and 520 nm (emission) was used to monitor these compounds.

Hypoxanthine/xanthine oxidase (HX/XO) was used to generate the superoxides. DHE (50 μM) was incubated with hipoxanthine (1 mM) and xanthine oxidase (0.05 units/ml) in PBS containing diethylenetriaminepentaacetate (100 μM). The mixture was stirred several times in air. After 60 min, the mixture was extracted using a spin column (MonoSpin C_18_, GL Sciences, Tokyo, Japan). The extraction procedure was performed according to the manufacturer’s instructions. The sample (700 μL) was poured into the pre-activated spin column, followed by centrifugation. The column was then washed with 500 μL of PBS by centrifugation. Finally, the oxidation products were eluted with 100 μL of CH_3_CN by centrifugation. Centrifugation was performed at 10,000 g for 1 min in all cases. The oxidation products of DHE mixed with PpIX or quercetin and irradiated with X-ray at 10 Gy and 30 Gy were also extracted using the spin column.

Fenton’s reaction was used to generate hydroxyl radicals and APF (5 μM) was incubated using iron (II) perchlorate (50 μM) and hydrogen peroxide (1 mM). A volume of 50 μL of oxidation products of APF was injected into the HPLC system without extraction.

### Cell viability assay

B16-BL6 mouse melanoma cells was supplied by the Riken Cell Bank (Tsukuba, Japan). The WST-8 cell viability assay was performed to assess cellular responses to XRS treatment and X-ray irradiation. WST-8 was used as described by the manufacturer. Prior to X-ray exposure, B16-BL6 cells were seeded into 96-well plates with a seeding density of 100 cell/well and incubated for 24 h. PpIX, quercetin, S1 or S6 was added 3 h before X-ray irradiation in 100 μL of culture medium at concentrations of 0, 1, and 5 μM/mL (0.25% DMSO). The plates were irradiated at a dose of 0, 1, 3, and 5 Gy. Immediately after irradiation, the medium was removed, washed with PBS, and 100 μL of fresh medium was added. 72 h after irradiation, 10 μL WST-8 was added to each well. The plates were incubated in a 5.0% CO_2_ humidified incubator at 37 °C for 1 h, following by measurement of the absorbance at 450 nm against a referenced absorbance at 600 nm using a plate reader. Relative cell viability was defined as the dye absorption ratio of treated versus untreated cells.

### Statistics

All numerical values are represented as the mean ± S.D. The mean values of the fluorescence intensities of ROS detection reagents correspond to the generated amount of ROS. Data for cell viability were analyzed using one-way analysis of variance (ANOVA). The Tukey–Kramer HSD test was used for post hoc pairwise comparison. Differences were considered statistically significant at p < 0.05.

## Supplementary information

dataset 1ABCD, 2,3,4,5,6
