# Supplementary material for: Screening of X-ray responsive substances for the next generation of radiosensitizers
Source: Sci Rep. 2019 Dec 3;9:18163. doi: 10.1038/s41598-019-54649-2 (PMC6890787; doi:10.1038/s41598-019-54649-2)
Supplement: Supplementary file 1 — dataset 1ABCD, 2,3,4,5,6 [file 41598_2019_54649_MOESM1_ESM.pdf]

Supplementary information

Title

Screening of X-ray responsive substances for the next generation of radiosensitizers.

Author list and affiliations

Akihiro Moriyama[1, 2], Takema Hasegawa [1, 2], Jiang Lei [1], Hitoshi Iwahashi[1], Takashi Mori[3], and Junko Takahashi* [1, 2]

1. The United Graduate School of Agricultural Science, Gifu University, 1-1 Yanagido, Gifu, Gifu 501-1193, Japan.
2. Molecular Composite Medicine Research Group, Biomedical Research Institute, National Institute of Advanced Industrial Science and Technology (AIST), 1-1-1 Higashi, Tsukuba, Ibaraki, 305-8566, Japan. Correspondence and requests for materials should be addressed to J. T. (email: junko-takahashi@aist.go.jp)
3. Animal medical Center, Gifu University, 1-1 Yanagido, Gifu, Gifu 501-1193, Japan.

(A)

Structure	Formula	MolWeight	Spectrum	Number
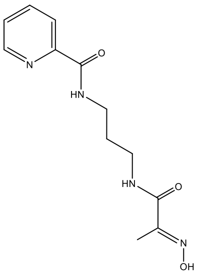	$C_{12}H_{16}N_4O_3$	264.285	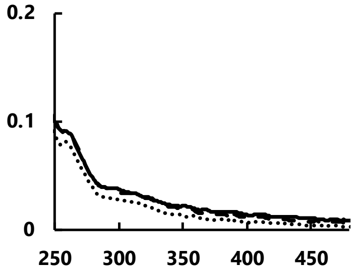	S1
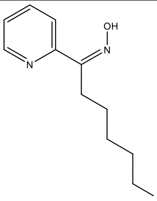	$C_{12}H_{18}N_2O$	206.289	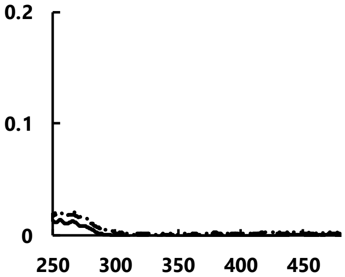	S2
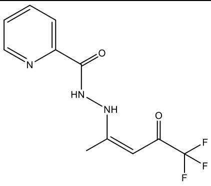	$C_{11}H_{10}F_3N_3O_2$	273.215	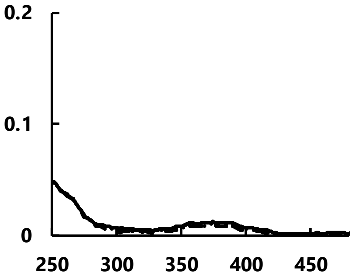	S3
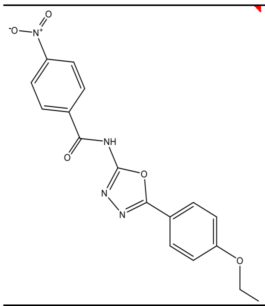	$C_{17}H_{14}N_4O_5$	354.322	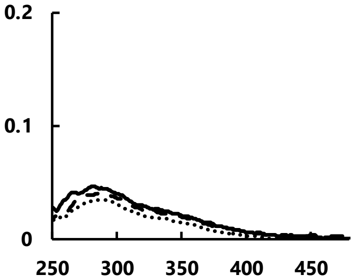	S4
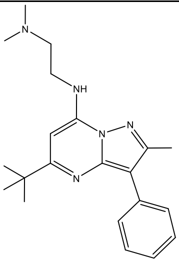	$C_{21}H_{29}N_5$	351.498	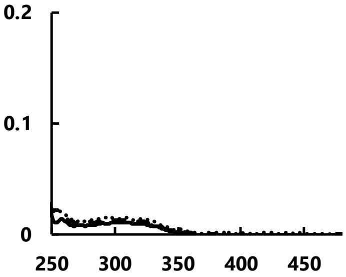	S5

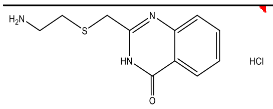	$C_{11}H_{14}ClN_3OS$	271.763	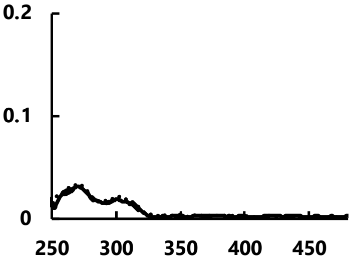	S6
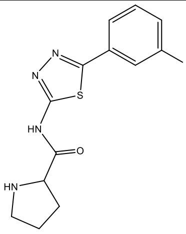	$C_{14}H_{16}N_4OS$	288.369	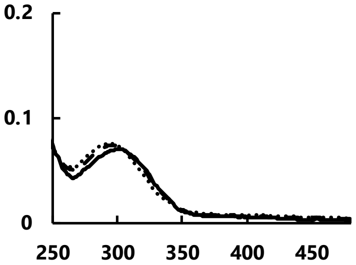	S7
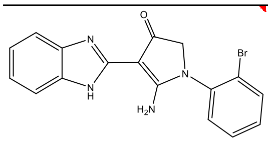	$C_{17}H_{13}BrN_4O$	369.222	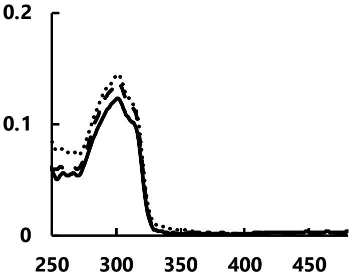	S8
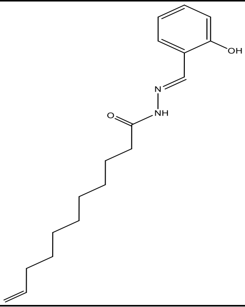	$C_{18}H_{26}N_2O_2$	302.418	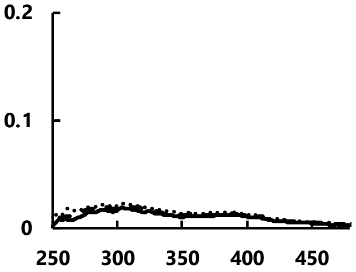	S9
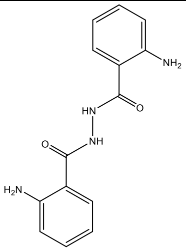	$C_{14}H_{14}N_4O_2$	270.292	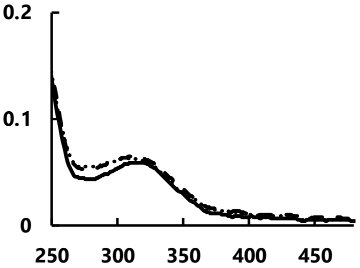	S10

(B)

Structure	Formula	MolWeight	Spectrum	Number
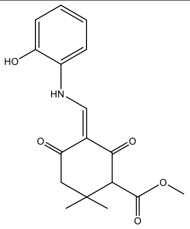	$C_{17}H_{19}NO_5$	317.341	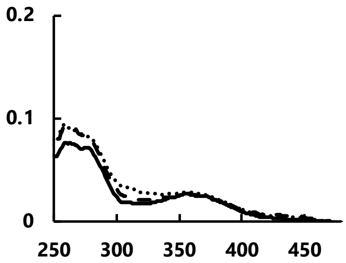	S11
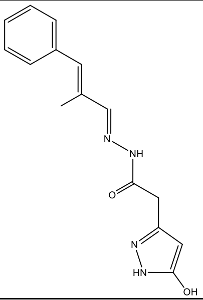	$C_{15}H_{16}N_4O_2$	284.319	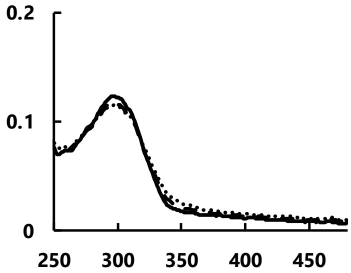	S12
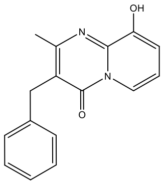	$C_{16}H_{14}N_2O_2$	266.3	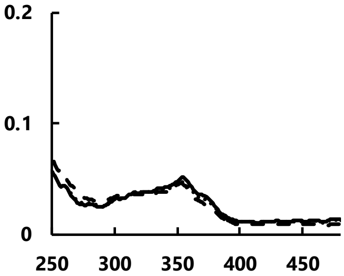	S13
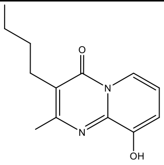	$C_{13}H_{16}N_2O_2$	232.283	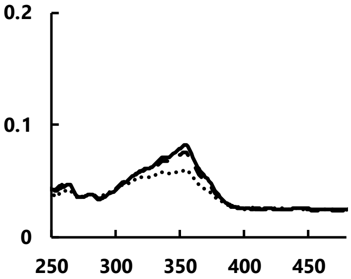	S14
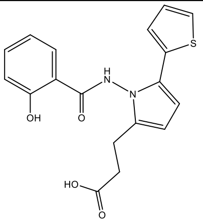	$C_{18}H_{16}N_2O_4S$	356.396	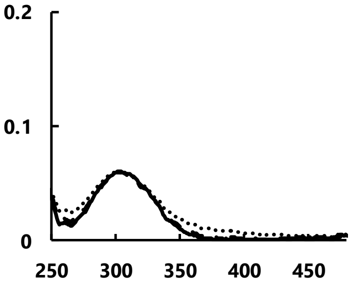	S15

(C)

Structure	Formula	MolWeight	Spectrum	Number
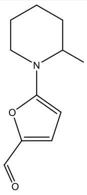	$C_{11}H_{15}NO_2$	193.246	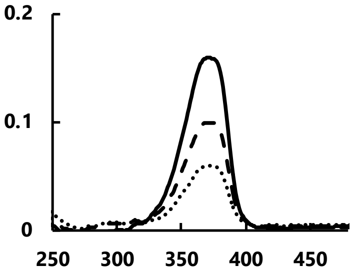	S16
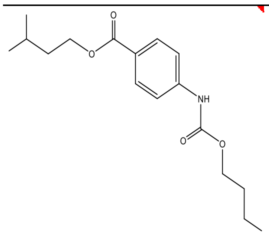	$C_{17}H_{25}NO_4$	307.39	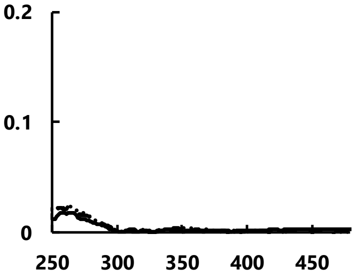	S17
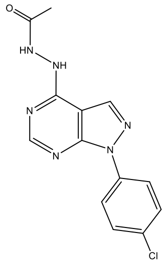	$C_{13}H_{11}ClN_6O$	302.722	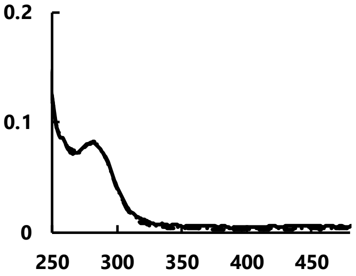	S18
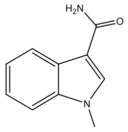	$C_{10}H_{10}N_2O$	174.203	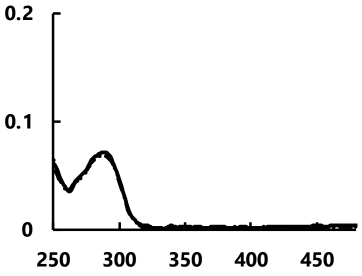	S19
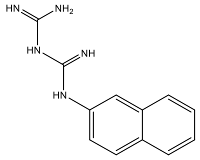	$C_{12}H_{13}N_5$	227.271	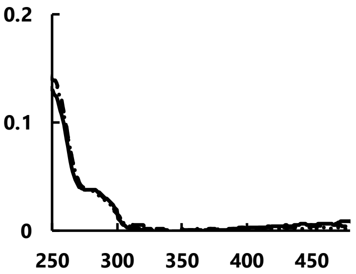	S20

(D)

Structure	Formula	MolWeight	Spectrum	Number
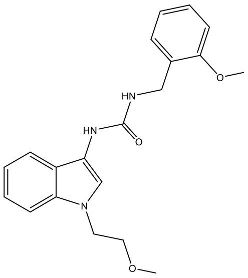	$C_{20}H_{23}N_3O_3$	353.422	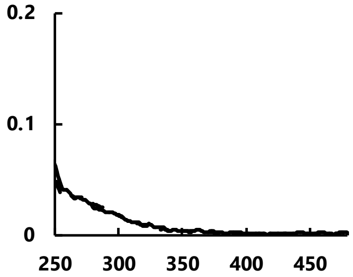	S21
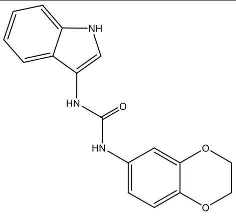	$C_{17}H_{15}N_3O_3$	309.325	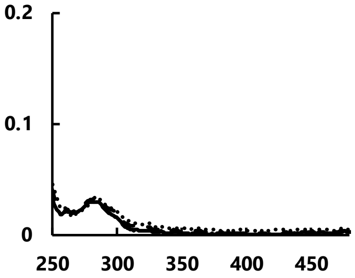	S22
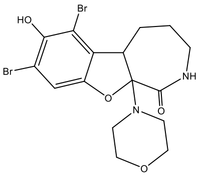	$C_{16}H_{18}Br_2N_2O_4$	462.138	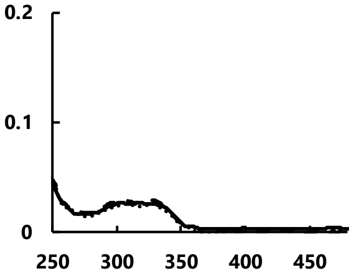	S23
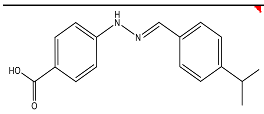	$C_{17}H_{18}N_2O_2$	282.343	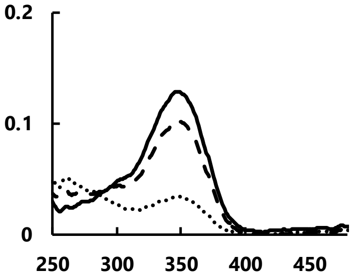	S24
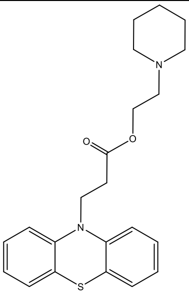	$C_{22}H_{26}N_2O_2S$	382.522	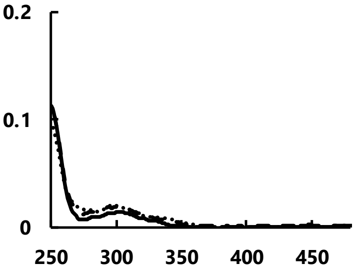	S25

Supplementary Fig. 1 The structural formula of the grouped hit compounds and the absorption spectra after X-ray irradiation.

Based on X-ray responses, we made 4 groups. Those that emit both superoxide and hydroxyl radicals are group1 (**Fig. S1A**) and those like quercetin that generate superoxides but eliminate hydroxyl radicals are categorized as group 2 (**Fig. S1B**). In addition, there is also a type that only generates hydroxyl radicals ((group 3, **Fig. S1C**)). Group 4 (**Fig. S1D**) includes compounds that substantially eliminate ROS, particularly hydroxyl radical when subjected to X-ray irradiation. Solid line represented the spectrum before X-ray irradiation, while dashed line and dotted line represented the spectrum after 10 or 30 Gy X-ray irradiation, respectively.

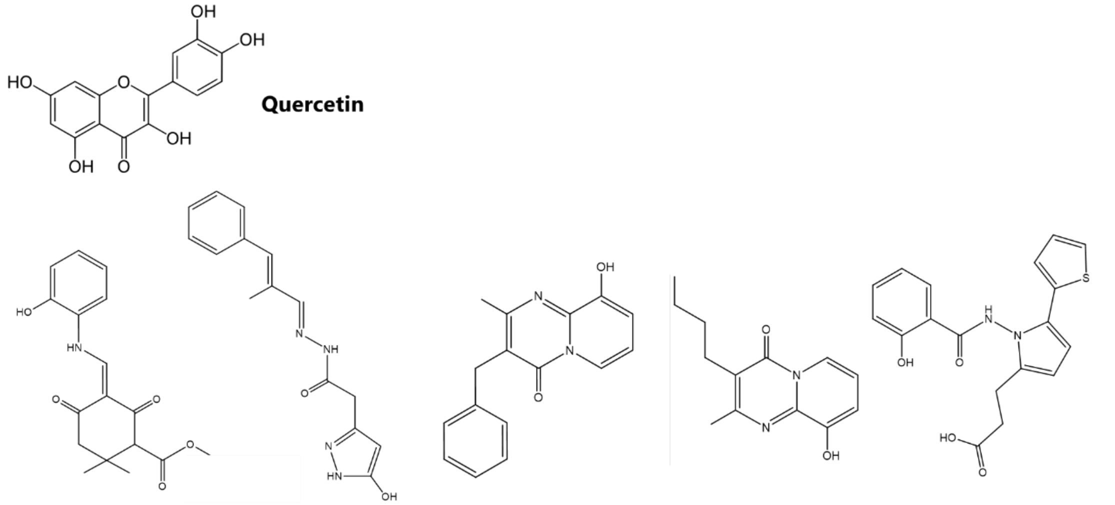

S11 S12 S13 S14 S15

Supplementary Fig. 2 Structural formula of quercetin and the compound assigned to group 2. The compounds those emit superoxides, but eliminate hydroxyl radicals are categorized as group 2. S13 and S14 have a structure which similar to that of quercetin. They exhibited similar behavior to quercetin as group2.

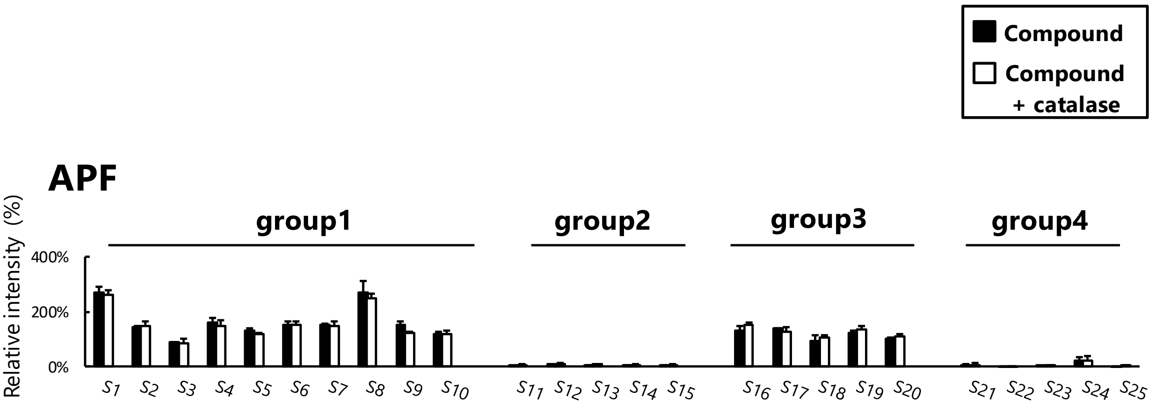

Supplementary Fig. 3 Relative fluorescence intensity of APF with each compound irradiated using X-rays with catalase.

Each compound was irradiated with 5 Gy X-ray undersame condition in Fig. 2 except catalase (100 Units/ mL) was challenged. Catalase was used as a quencher for hydroxyl radical for APF fluorescence since hydroxyl radical may have originated from H_2O_2 via superoxide dismutation.

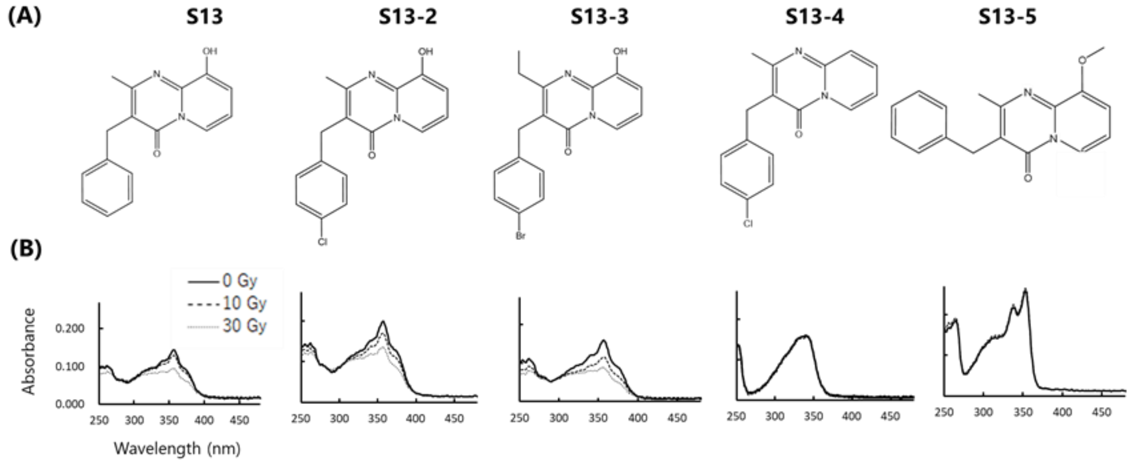

Supplementary Fig. 4 Spectra of S13 derivative compounds before or after X-ray irradiation (10 or 30 Gy).

(A) Structural formula of S13-derivative compounds (B) Spectra of S13-derivative compounds (final concentration of 5 μM, 0.25% DMSO) before or after X-ray irradiation (10 or 30 Gy). Solid line represented the spectrum before X-ray irradiation, while dashed line and dotted line represented the spectrum after 10 or 30 Gy X-ray irradiation, respectively.

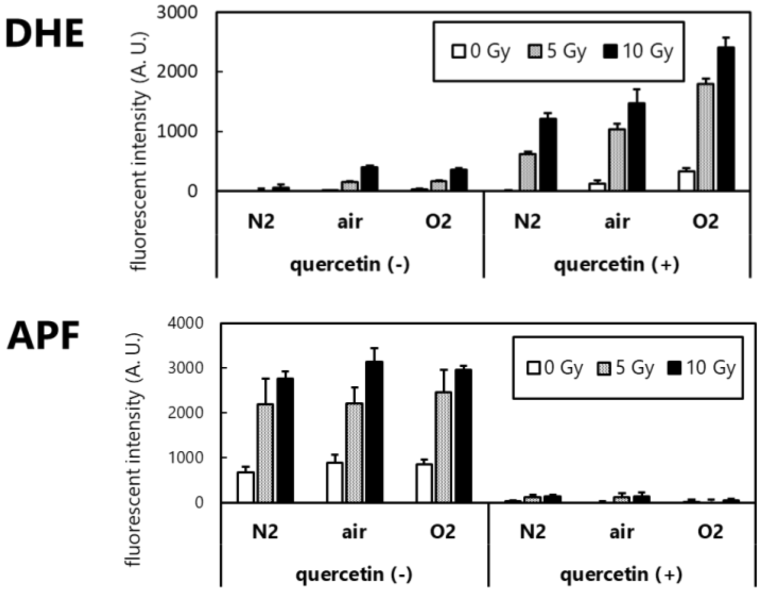

Supplementary Fig. 5 Evaluation of the effect of dissolved oxygen for ROS generation via the reaction with quercetin and X-ray irradiation.

(A): •OH measured by AFP. (B): $O_2^{\bullet -}$ measured by DHE. To evaluate the effect of dissolved oxygen for ROS generation, N_2, air, or O_2 gas was bubbled through the reaction mixture. A volume of 1 mL quercetin at 5 µM and 50 µM DHE or 5 µM APF were placed in gas-tight 5 mL containers with a gas inlet and outlet port. After N_2, air, or O_2 gas was bubbled through the containers for 2 minutes, the inlet and outlet valves were shut off, and X-ray irradiation was immediately performed. Subsequently, each mixture with DHE was measured by microplate reader at Em: 485 nm and Ex: 610 nm, mixture with APF was measured at Em: 480 nm and Ex: 520 nm.

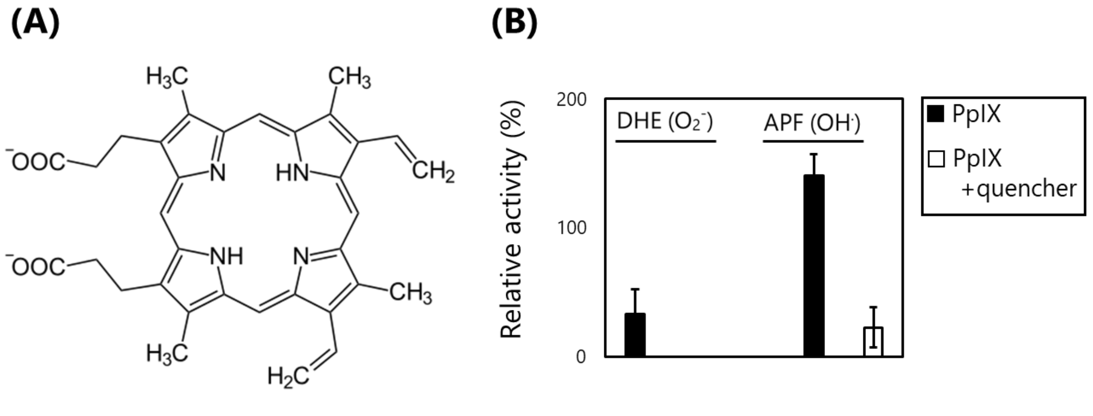

Supplementary Fig. 6 Properties and X-ray responsiveness of protoporphyrin (PpIX).

(A) Structural formula of PpIX (B) 5 µM PpIX (in 0.25% DMSO) was irradiated in the presence of either 50 µM DHE ($O_2^{\bullet -}$ assay assay) or 5 µM APF (•OH assay). Radiation dose was either 5 or 10 Gy. The assays were performed either in the absence or presence of ROS quenchers (DHE; 7.5 U SOD, APF; 10% ethanol)
